# Comparative analysis of CRISPR cassettes from the human gut metagenomic contigs

**DOI:** 10.1186/1471-2164-15-202

**Published:** 2014-03-17

**Authors:** Anna A Gogleva, Mikhail S Gelfand, Irena I Artamonova

**Affiliations:** N. I. Vavilov Institute of General Genetics, Russian Academy of Sciences, Gubkina str. 3, Moscow, 119991 Russia; A. A. Kharkevich Institute for Information Transmission Problems, Russian Academy of Sciences, Moscow, Russia; Faculty of Bioengineering and Bioinformatics, M. V. Lomonosov Moscow State University, Moscow, Russia

**Keywords:** CRISPR, Human gut, Microbiome

## Abstract

**Background:**

CRISPR (**C**lustered **R**egularly **I**nterspaced **S**hort **P**alindromic **R**epeats) is a prokaryotic adaptive defence system that provides resistance against alien replicons such as viruses and plasmids. Spacers in a CRISPR cassette confer immunity against viruses and plasmids containing regions complementary to the spacers and hence they retain a footprint of interactions between prokaryotes and their viruses in individual strains and ecosystems. The human gut is a rich habitat populated by numerous microorganisms, but a large fraction of these are unculturable and little is known about them in general and their CRISPR systems in particular.

**Results:**

We used human gut metagenomic data from three open projects in order to characterize the composition and dynamics of CRISPR cassettes in the human-associated microbiota. Applying available CRISPR-identification algorithms and a previously designed filtering procedure to the assembled human gut metagenomic contigs, we found 388 CRISPR cassettes, 373 of which had repeats not observed previously in complete genomes or other datasets. Only 171 of 3,545 identified spacers were coupled with protospacers from the human gut metagenomic contigs. The number of matches to GenBank sequences was negligible, providing protospacers for 26 spacers.

Reconstruction of CRISPR cassettes allowed us to track the dynamics of spacer content. In agreement with other published observations we show that spacers shared by different cassettes (and hence likely older ones) tend to the trailer ends, whereas spacers with matches in the metagenomes are distributed unevenly across cassettes, demonstrating a preference to form clusters closer to the active end of a CRISPR cassette, adjacent to the leader, and hence suggesting dynamical interactions between prokaryotes and viruses in the human gut. Remarkably, spacers match protospacers in the metagenome of the same individual with frequency comparable to a random control, but may match protospacers from metagenomes of other individuals.

**Conclusions:**

The analysis of assembled contigs is complementary to the approach based on the analysis of original reads and hence provides additional data about composition and evolution of CRISPR cassettes, revealing the dynamics of CRISPR-phage interactions in metagenomes.

**Electronic supplementary material:**

The online version of this article (doi:10.1186/1471-2164-15-202) contains supplementary material, which is available to authorized users.

## Background

Prokaryotic cells inhabiting the human body outnumber its own eukaryotic cells at least ten to one, with the overwhelming majority of bacteria residing in the intestine. This complex community of symbiotic, pathogenic and commensal microorganisms is called the microbiome [1]. The human gut microbiome might be considered as an organ within an organ [2]. It has been shown to be indispensable for the human life as it is capable of vitamin production [3, 4], digestion of complex polysaccharides [5], controlling intestinal epithelial cell proliferation through the production of short-chain fatty acids [6], and influencing the normal development and function of the mucosal immune system [7]. While bacteria are responsible for these functions, bacteriophages, in turn, influence their abundance in the human gut [8, 9].

As a reaction to an ongoing phage pressure, prokaryotes have developed numerous defence mechanisms [10]. The CRISPR systems are especially interesting, as they retain the history of interactions between viruses and their prokaryotic hosts [11–13]. Despite being highly diverse [14], typically they are comprised of a CRISPR cassette containing an array of unique spacer sequences (25–70 bp) alternating with conserved short direct repeats and preceded by a 5′-leader sequence of 200–500 bp. The systems also include numerous CRISPR-associated (*cas*) genes that encode proteins performing various, only partially characterized functions essential for the system’s activity [15]. A CRISPR cassette is transcribed as a long precursor RNA molecule that is further cleaved into small fragments (crRNA), each containing one complete spacer. crRNAs are the main players in the RNA-guided degradation of foreign replicons [11, 16, 17]. Accumulation of new spacers occurs at one side of a cassette, adjacent to the leader sequence, while internal spacers may be deleted by recombination. Hence older spacers are shifted to the 3′-end, and cassettes retain a unique chronological footprint of viruses that have infected a given strain [18–20]. However, a part of a cassette or even a complete cassette may be also acquired via horizontal gene transfer [21]. In addition, identical or similar repeats in different CRISPR cassettes may indicate their common ancestry.

Up to 60% of all microorganisms that inhabit the human body are considered to be unculturable [22]. Culture-independent metagenomics is the most powerful approach to study the composition and dynamics of complex microbial communities. The metagenomics data allow one to obtain a complete snapshot of coexisting microorganisms, both prokaryotes and their viruses.

To date, CRISPR systems have been analyzed in metagenomic datasets of several environments including acidophilic biofilms [23], acidic hot springs in Yellowstone National Park [24], Australian hypersaline Lake Tyrrell [25], ocean metagenome produced by the Global Ocean Sampling (GOS) expedition [26], and the rumen microbiome [27].

A considerable effort is directed to large-scale investigation of the human microbiome using the metagenomic approach. The main aim of these studies is to understand the role of the endogenous flora in health and disease. Among all body sites, the diversity of microorganisms in the human gut is known to be the highest [28]. The data from several human microbiome metagenomic projects are available, as well as human gut virome data [29–34]. A high level of microbial diversity and availability of metagenomic datasets obtained using various sequencing techniques make the human gut microbiome a promising object for studying CRISPR systems.

Indeed, CRISPR cassettes were characterized across body sites in different individuals through independent projects [35–37] and as a part of the Human Microbiome Project [38] with a particular attention to the gut metagenome [38–40]. In these studies, raw reads containing CRISPR repeats were collected, followed either by the analysis of the spacer content [39] or reassembly of repeat-containing reads into contigs [38]. This approach allowed the authors to identify thousands of spacers, although it was limited to CRISPR cassettes with already known repeats. While being a powerful tool to study the distribution of spacers, this strategy does not account for the CRISPR cassette structure, and hence may not track the evolutionary dynamics of spacers within cassettes. To offset this, we identified cassettes in assembled contigs. The comparison of spacers and repeats of these cassettes with previously analyzed spacers and repeats, in particular, those identified by read-based techniques, yielded only few matches. This suggests that both approaches are useful as they produce complementary findings. We analyzed the CRISPR content in three human gut metagenomes, two of which have not been analyzed earlier in this context. We identified CRISPR cassettes, compared the sets of repeats and spacers with the ones identified in earlier studies and analyzed the differences, identified protospacers, reconstructed the taxonomy distribution of cassettes and protospacers, characterized the distribution of spacers and protospacers in individual metagenomes, and, finally, described the dynamics of spacer positions within CRISPR cassettes for different classes of spacers.

## Methods

### Metagenomic datasets

#### Human microbiomes

The gut samples of the **H**uman **M**icrobiome **P**roject (**HMP**) dataset were downloaded as an assembly in 1,889,651 contigs [41]. The total length of HMP contigs comprised 3,732 Mb. The fecal DNA samples were collected from 124 adults of various ages (18–69) sequenced by Illumina GA machines [28].

The assembled metagenomic dataset from 13 healthy Japanese individuals (**JPN**) was downloaded from the CAMERA website [42]. This dataset contained 353,805 contigs of the total length 463 Mb. The samples were collected from adults and children including unweaned infants (6 months to 45 years), comprising two families of three and four members, and six unrelated individuals. The shotgun reads were obtained using MegaBACE4500 sequencers (GE Healthcare) [43].

The contigs from the **D**istal **G**ut metagenomic project (**DG**) were downloaded from the NCBI website [44]. The assembly contained 22,508 contigs, comprising 336 Mb. The reads were sequenced using the ABI 3730xl DNA analyzer [45].

The information about metagenomic datasets used here is summarized in Table 1.

**Table 1:** **Characteristics of the analyzed metagenomic datasets**

Metagenomic project	Number of contigs/reads	Total length	Source	Individuals involved	Sequencing platform	Assembly algorithm
The Human Microbiome Project **(HMP)**	1,889,651 contigs	3,732 Mb	Fecal samples	124 Europeans of various ages (18–69)	Illumina GA	MetaMos
		(N50 = 3692)				
Healthy Human Gut Metagenomes **(JPN)**	353,805 contigs	463 Mb	Fecal samples	13 Japanese individuals (6 months – 45 years), comprising 2 families (of 3 and 4 members) and 6 unrelated individuals	MegaBACE4500 sequencer (GE Healthcare)	PCAP
		(N50 = 1180)				
Distal gut metagenomic project **(DG)**	22,508 contigs	336 Mb	Fecal samples	2 healthy adults	ABI 3730x1 DNA analyzer	Celera Assembler
		(N50 = 1657)				

### Identification and analysis of CRISPR cassettes

To construct a set of CRISPR cassettes for each metagenomic dataset, we used three algorithms, PILER-CR [46], the CRISPR Recognition Tool (CRT) [47], CRISPRFinder [48], and a previously designed filtering procedure [26].

In addition, we attempted to use Crass [49] with default parameters to assemble CRISPR cassettes from metagenomic reads. While the number of detected cassettes for the DG read dataset was comparable to that obtained by our procedure, Crass did not assemble CRISPR cassettes from the HMP data. Relaxing parameters for the number of repeats and spacer or repeat length (-n 2 -w 6 -s 20 -S 55) allowed Crass to identify only one CRISPR cassette in the HMP dataset. For the JPN dataset metagenomic reads were not available, and hence Crass could not be applied. Hence, for uniformity, Crass predictions were not considered further.

To determine contig taxonomy, contigs were subjected to the BLASTX search [50, 51] against the non-redundant protein collection (NR) of GenBank [52] (*e*-value threshold 1e^-6^). Taxonomic labels were assigned manually based on the degree of consistency in the taxonomy origin of the top hits. Taxonomic labels at the phylum level were assigned if at least top ten hits belonged to one phylum; taxonomic labels at the level of class, family, and genus were assigned if the majority of top 30 hits belonged to the same taxon of that level. If top hits were taxonomically diverse, the contig was assigned with a nonspecific taxonomic label. A contig might not be assigned with a taxonomic label for the following reasons: (1) CRISPR cassette covering entire contig length; (2) CRISPR cassette flanked by regions containing only universal *cas* genes, known to be subject to frequent horizontal gene transfer, so that their phylogeny does not necessarily reflect taxonomy [53]; (3) flanking regions containing genes with no significant similarity to any entry in the non-redundant GenBank collection. The *cas* genes were identified as described previously [26].

We considered two types of data to perform the BLASTN search in order to identify sources of spacers (protospacers). Firstly, we compared spacer sequences to all viral entries, including complete genomes, from GenBank. Secondly, we compared the spacer sets with the human metagenomic datasets themselves, assuming that these data may still contain contigs of phage, prophage, or plasmid origin after filtering out small particles (according to the metagenome DNA isolation protocol [54]).

If a mismatch between two similar sequences is located at a distance less than one word from the sequence end, BLASTN would not extend the alignment over this mismatch. Since the alignments between spacers and protospacers are necessarily short, this means that spacer-protospacer pairs with mismatches in the middle might be aligned by BLASTN only partially. To offset this and to estimate the real number of mismatches in identified spacer-protospacer pairs, all obtained hits were postprocessed, and if the observed matching regions were shorter than the corresponding spacer sequence they were extended in one or both directions to match the full length of the spacer.

The number of mismatches was calculated for extended alignments of spacer-protospacer pairs and a threshold of four mismatches along the entire spacer length was set to define candidate protospacer sequences. To ensure that a sequence matched by a spacer is not an undetected CRISPR cassette, we performed a parallel BLASTN search for repeat sequences from the corresponding CRISPR cassettes against the same datasets as it was done for spacer sequences.

The taxonomic labels were assigned to protospacer-containing contigs as described above, and then transferred to the respective spacers as follows: if the protospacer was of a phage or plasmid origin, we used the taxonomical information about its host, whereas if the protospacer came from a sequence of a bacterial origin, its taxonomy was assigned as described above. If the spacer was already assigned with a taxonomical label, the assignments were compared.

In order to estimate the significance of the observed similarities between spacers and a sequence database, we generated randomized sets of “pseudospacers”, where each spacer was replaced by a random fragment of the same length and, if possible, from the same contig. The range for randomization excluded regions covered by CRISPR cassettes. If a CRISPR cassette covered a metagenomic contig (almost) completely, i.e., if both flanking sequences were shorter than 100 nt, a fragment of the same length was taken from a randomly selected contig coming from the same individual but not containing predicted CRISPR cassettes.

However, a straightforward application of this procedure would be confounded by gene homology. This would manifest as similarity extending beyond the pseudospacer-pseudoprotospacer pair. To avoid this possibility, we extracted the flanking sequences of selected pseudospacers. These sequences had the same length as the repeats in the respective cassette. They were run against the same datasets as pseudospacers. A pseudospacer-pseudoprotospacer pair was taken into account only if none of its pseudospacer-flanking sequences matched the same sequence.

Repeat clusters were constructed as described previously [26] using the standard BLASTCLUST procedure applied to the set of consensus repeat sequences (parameters: -L 0.5 -S 50 -e F -p F -W 15). All clusters with more than one member were collected. Alignments for the obtained clusters were constructed using the standard MUSCLE procedure [55]. For further analysis, the repeats were considered to be similar if they belonged to the same repeat cluster.

To search for PAM sequences (**p**rotospacer **a**djacent **m**otifs), 10 nt regions flanking protospacers from both sides were used [56].

CRISPR cassettes were oriented, when possible, according to the position and direction of transcription of *cas* genes. An end of a cassette was labeled as the leader terminus if the adjacent region of the contig contained a *cas* gene in the proper orientation. In addition, cassettes lacking flanking sequences of sufficient length were oriented by comparison to cassettes from the same repeat cluster, for which the orientation had been already assigned, assuming that the repeat should be encoded on the same strand for the entire cluster. For cassettes not belonging to clusters with defined orientation, the leader and trailer termini could not be determined, and such cassettes were not considered in the orientation-dependent analyses.

*Targeting spacers* were defined as spacers having at least one reliable protospacer in the same individual metagenome. *Shared spacers* were defined as spacers observed in two or more individual metagenomes.

To estimate whether targeting spacers tend to occur close to the leader-end of cassettes, and shared spacers, to the trailer-end, the following Monte-Carlo simulation was implemented. Only complete cassettes (with non-cassette flanking sequences) with defined orientation, were used. Spacers in each cassette were enumerated. For each cassette, serial numbers of all targeting (resp. shared) spacers in the cassette were summarized. Then the obtained statistics were summarized for the whole set of considered CRISPR cassettes. Hence, we obtained a single value equal to the sum of all serial numbers of all targeting (resp. shared) spacers. After that, spacers in each cassette were randomly shuffled and the same procedure was applied. It was repeated 100,000 times and the distribution of the analyzed statistic was built for the targeting and shared spacers. Then the statistic obtained for real cassettes was compared with the constructed distributions, and the *p*-values were calculated.

To check whether spacers and protospacers tend to co-occur, we performed the following test. For each individual we constructed a 2 × 2 contingency table featuring the number of spacer-protospacer pairs with spacer (resp. protospacer) coming from this individual. For the resulting set of contingency tables, the Cochran–Mantel–Haenszel (CMH) statistic was calculated [57]. As a null hypothesis we assumed that the occurrences of spacer and corresponding protospacer in an individual are independent. To check whether our data fit this hypothesis, we shuffled protospacers across the individual metagenomes, so that the number of protospacers in a given individual remained unchanged. This procedure was performed 10,000 times, and in each round of permutations the CMH statistic was calculated. The obtained distribution was used to estimate the *p*-value of the observed statistic.

## Results and discussion

### Characteristics of CRISPR cassette sets

We used three publicly available human metagenomes to search for CRISPR cassettes. The latter were identified by several existing programs (see Methods). To exclude false predictions of CRISPR cassettes in the metagenomic data, a filtering procedure was applied [26]. This procedure retains the following types of cassettes: (1) cassettes predicted by the CRT, CRISPRFinder and PILER-CR programs simultaneously; (2) candidate cassettes (cassettes predicted by only one or two programs listed) adjacent to *cas* genes; (3) candidate cassettes whose repeat consensus is similar to the repeat consensus of a cassette already accepted based on (1) or (2).

**Figure 1:**
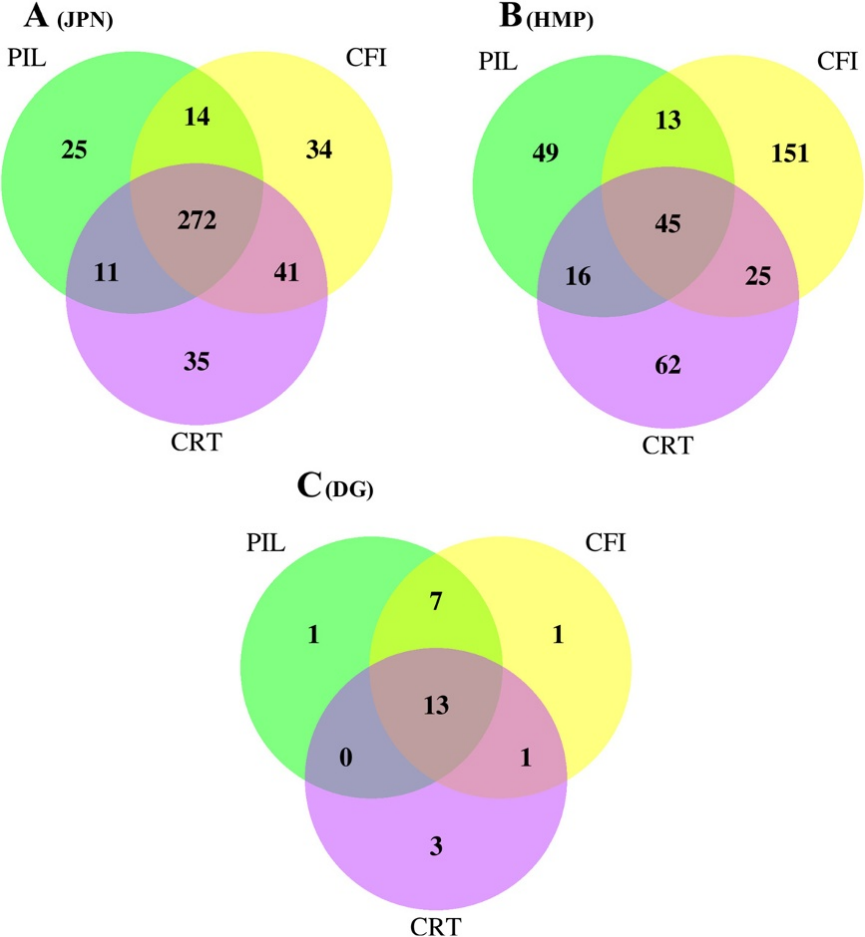
**Venn diagram showing the numbers of CRISPR cassettes identified by the three programs.** JPN **(A)**, HMP **(B)**, and DG **(C)** metagenomes are presented. Abbreviations for the program names are: CRT – the CRISPR recognition tool; CFI – CRISPRFinder; PIL – PILER-CR.

The sets of identified cassettes are shown in Figure 1 and characterized in Table 2 and Additional file 1: Table S1. The largest set of cassettes was identified in the JPN dataset, followed by HMP, and few cassettes were observed in the DG metagenome. Among the algorithms, the largest number of candidate cassettes was produced by CRISPRFinder, followed by CRT and PILER-CR, with considerable overlap between the predictions (Figure 1). Examination of individual predictions demonstrated that CRT and PILER-CR tend to consider genomic repeats and low-complexity regions as a candidate CRISPR cassette, whereas CRISPRFinder reports numerous short cassettes of the type “repeat-spacer-repeat”.

**Table 2:** **Statistics of identified CRISPR cassettes and spacers**

Metagenome dataset	JPN	HMP	DG
**Cassettes**			
**Identified by:**			
**PILER-CR**	322	121	17
**CRT**	359	149	21
**CRISPRFinder**	361	235	22
**All three programs**	272	45	13
**1 or 2 programs, but adjacent to** ***cas*** **genes**	24	33	1
**Final set of cassettes:**			
**Total number**	296	78	14
**Cassettes adjacent to** ***cas*** **genes**	70 (24%)	56 (71%)	6 (43%)
**Cassettes with assigned taxonomy**	73 (25%)	69 (82%)	9 (64%)
**Cassettes with assigned CRISPR-cas type:**			
**I type**	18 (6%)	16 (20%)	1 (7%)
**II type**	9 (3%)	4 (5%)	1 (7%)
**III type**	6 (2%)	18 (23%)	1 (7%)
**Spacers**			
**Total number**	3410	378	175
**Unique spacers**	2992	352	174
**Spacers with protospacers in:**			
**The same metagenomic dataset**	136	59	0
**NR database**	17	9	0
**Repeats**			
**Unique**	170	74	11
**Repeats with matches in CRISPRdb**	23	0	0
**Repeats from known clusters according to the CRISPRmap algorithm**	122	18	8

Columns correspond to three metagenome datasets.

Then, we considered candidate CRISPR cassettes identified by at least one of the programs, and adjacent to *cas* genes (Table 2). There are several possible reasons why these cassettes have not been identified initially, including short cassette lengths, varying length of spacers, divergent repeats, *etc*., confounding individual programs. Notably, of 132 cassettes adjacent to *cas* genes, 119 (90%) had repeats not observed in other databases or complete genomes. That done, no candidate cassettes with repeats similar to the repeats of already accepted cassettes were observed. Hence, filtering condition (3) turned out to be redundant. This proves robustness of the procedure.

The final set of CRISPR cassettes consisted of 298, 78, and 14 cassettes from the JPN, HMP, and DG metagenomes, respectively. Two distinct cassettes were never observed in one contig. In all three metagenomic datasets, a considerable fraction of cassettes were adjacent to putative *cas* genes. We detected 70, 56, and 6 such cassettes in the JPN, HMP, and DG metagenomes, respectively, comprising 24%, 71%, and 43% of the total cassette number in the respective set.

The set of 298 CRISPR cassettes in the JPN metagenome contained 3410 spacers, comprising 2992 unique spacers. 378 spacers from 78 HMP cassettes comprised 352 unique ones. Only one spacer out of 175 spacers found in 14 DG cassettes occurred twice (Table 2). The non-redundant set of repeat sequences contained 170, 74, and 11 unique repeats for the JPN, HMP, and DG metagenomes, comprising 139 repeat clusters (Table 2).

Once a reliable set of CRISPR cassettes was constructed, we compared consensus repeat sequences from these cassettes (Additional file 1: Table S1) with repeats from already known CRISPR cassettes deposited in CRISPRdb [58]. Only 23 of 255 identified unique repeat sequences matched repeats from the CRISPRdb database. All matched repeats originated from the JPN metagenome and corresponded to 17 repeat clusters. Such a small intersection with CRISPRdb indicates that most CRISPR cassettes identified here are novel.

Generally, two different approaches to identification of CRISPR cassettes in metagenomic data are feasible: making prediction on assembled contigs or extracting spacers directly from raw reads. CRISPR prediction on assembled contigs retains the order of spacers in a cassette. On the other hand, assembly of sequences containing repeats is difficult, and hence a considerable fraction of CRISPR cassette-containing reads would remain unexplored. The other approach, recently used to identify CRISPR cassettes in the human gut metagenome [39], analyzes raw reads and extracts spacer sequences flanked by sufficiently long repeat segments from already known CRISPR cassettes from existing databases. Here, the set of identifiable spacers is limited by the set of known repeats. A combination of the described strategies, named “targeted assembly of CRISPR cassettes”, first selects reads matched by known repeat sequences or predicted by a program (CRT), and then reassembles them into CRISPR cassettes [38]. We compared our results with those produced by these two approaches on the human gut metagenomic data.

The CRISPR set identified by Stern *et al*. [39] in raw HMP reads contains 52,267 spacers, 48,484 of which are unique. Comparing these spacers with the spacer sets identified here, we found only 15 matches in our set of the HMP spacers (originating in four different cassettes; only three spacers from the Stern set exactly matched spacers from the HMP set) and 125 matches in the JPN set (originating in 40 different cassettes). No matches with spacers from the DG set were observed. The matched spacers comprise 3% and 4% of our unique spacers in the HMP and JPN sets, respectively, i.e., roughly the same fractions of the whole sets of unique spacers.

The fact that only few of the spacers from Stern *et al.*[39] matched spacers identified here could be caused by two reasons: either these spacers or the respective cassettes were present in the HMP assembled contigs, but had been missed by our identification procedure, or the reads containing these spacers were not assembled in the contigs. To distinguish between these alternatives, we performed BLASTN search for unique spacers from the Stern set against all assembled HMP contigs. Most spacers (39,273, 81%) did not match contigs. The remaining spacers and the matching contigs were analyzed in more detail. As repeats identified by Stern *et al*. were not available, we checked whether HMP contigs matched by the Stern spacers contained repeats from the HMP repeat set (identified here) and/or known CRISPR repeats from CRISPRdb [58]. With the exception of the contigs with three spacers that exactly matched HMP spacers identified here and twelve spacers with non-exact matches (up to four mismatches), none of contigs matched by the Stern spacers contained repeats either from CRISPRdb or from our HMP repeat set. Further, six spacers from the Stern set matched HMP contigs with questionable (in the CRISPRFinder notation) CRISPR cassettes, which had been predicted by only one or two algorithms due to a low level of repeat conservation and varying spacer lengths within a candidate cassette. This strongly suggests that we did not miss any considerable number of identifiable cassettes with spacers from the Stern set while the false negative rate of our predictions given the available data is low. On the other hand, a considerable fraction of the Stern spacers (9,100, 19%) matched HMP contigs without repeats or CRISPR cassettes predicted by either algorithm. Given that the Stern *et al.*[39] procedure relies on known repeats, these matches could be protospacers of those spacers.

The set of CRISPR cassettes identified in the HMP data by the targeted assembly approach contained 150 cassettes, 86 of which were found in gut samples [38] and we used the latter for further analysis. Comparison of these data with the repeat sequences identified here yielded only four matches with repeats from the HMP set, originating in four different CRISPR cassettes. Only 25 of HMP repeats identified by Rho *et al.*[38] had matches in the assembled HMP contigs. These contigs did not pass our filters as they did not contain CRISPR cassettes identified by the three programs due, in particular, to short cassette length or degenerate repeats; neither they contained *cas* genes. As in the previous case, it shows that the Rho cassettes absent in our set have been produced by reads that had not been assembled into contigs.

Unfortunately, we could not make a universal comparison as the necessary data were not available – only spacers were provided in [39] and only repeats, in [38]. Still, this analysis shows that CRISPR cassettes identified by our approach are mostly novel and considerably different from CRISPR cassettes found in human gut microbiomes earlier, and hence the contig-based and read-based approaches produce complementary results. Indeed, the read-based approach missed cassettes with new repeats, while the contig-based techniques missed cassette fragments in unassembled reads.

The number of CRISPR cassettes in individual metagenomes varied. No dependence between the number of identified cassettes or spacers and the average contig size or sample size could be observed (Additional file 2: Figure S1); however, the sequencing technologies and assembly algorithms could be responsible for the observed differences between the datasets. On the other hand, the number of identifiable CRISPR cassettes might reflect the major taxonomic breakdown of human gut microbiota and, indirectly, enterotypes of particular individuals [59].

### Taxonomy of metagenomic contigs containing CRISPR cassettes

To define the taxonomic origin of contigs containing the identified cassettes, a BLASTX-based procedure was used (for details see Methods). The short length of metagenomic contigs combined with the propensity of *cas* genes to horizontal gene transfer makes taxonomic predictions for CRISPR-containing contigs difficult. We assigned the taxonomy at least at the domain level to 73 of 296 JPN cassettes (25%), 69 of 78 HMP cassettes (82%), and 9 of 14 DG cassettes (64%). The differences in the average fractions of CRISPR-containing contigs with assigned taxonomy reflect the average lengths of the contigs in the respective samples.

**Figure 2:**
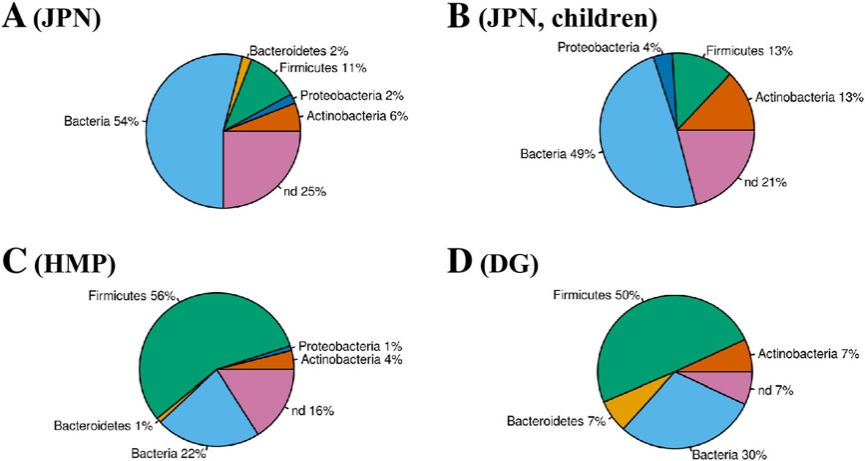
**Taxonomy of CRISPR-containing contigs.** The presented metagenomes are: JPN **(A)**; JPN, children only **(B)**; HMP **(C)**; DG **(D)**. The abbreviation “nd” stands for “not determined”.

Despite the fact that the total number of cassettes identified in the three studied metagenomes was considerably different, the prevalent taxa of contigs with CRISPR cassettes were similar in all three datasets (Figure 2). The largest fraction of contigs with assigned taxonomy belonged to Firmicutes. We observed 33, 43, and 8 contigs of the Firmicutes origin in the JPN, HMP, and DG metagenomes, respectively, with the majority of them belonging to Bacilli and Clostridia. In the JPN sample, 20 contigs were observed in adults and 13, in children.

The second major group in the JPN metagenomic dataset was comprised of 19 contigs (5 in adults and 14 in children) from Actinobacteria with a majority of them assigned to the *Bifidobacterium* genus. Contigs belonging to Proteobacteria and Bacteroidetes/Chlorobii group comprised about 2% each. Proteobacterial contigs were predominantly coming from Enterobacteriaceae, in particular, from *Escherichia coli.* For about 25% of JPN contigs, no strong evidence of any particular bacterial phylum was detected, so these contigs were generically assigned to Bacteria (Figure 2A, B). According to a previous analysis of the JPN metagenome [43], the major constituents in adults and children are different. CRISPR-containing metagenomic contigs originating from children were assigned to the same predominant taxa as the whole dataset, but the fraction of contigs assigned to Actinobacteria was larger (13%) (Figure 2B).

As mentioned above, the largest fraction of HMP CRISPR-containing contigs (56%) was assigned to Firmicutes (Figure 2C). 22% of HMP contigs showed a clear bacterial origin but could not be assigned to any particular phylum, while for 16% of contigs, the taxonomic origin could not be determined. A minority of CRISPR-containing contigs in the HMP metagenome originated in Actinobacteria, Bacteroidetes, and Proteobacteria, together comprising less than 6%. In the DG metagenomic dataset, one cassette was assigned to Bacteroidetes and one, to Actinobacteria (Figure 2D). No archaeal contigs containing CRISPR cassettes were observed in any human gut metagenome.

The taxonomy breakdown of the CRISPR-containing contigs slightly differs from that based on 16S rRNA for the entire metagenomic datasets. According to 16S rRNA, the major constituents of the JPN metagenome in adults and weaned children were always Bacteroidetes followed by several Firmicutes genera and the genus *Bifidobacterium.* In the case of infants, representatives of Bifidobacteriaceae and Enterobacteriaceae were predominant [43]. This does not agree with the prevalence of Firmicutes CRISPR-containing contigs in the JPN metagenome*.* In HMP, the major fraction of the microbial diversity, according to 16S rRNA, is comprised by Firmicutes, followed by almost equal fractions assigned to Bacteroidetes, Actionobacteria and Proteobacteria*,* i.e., it is very similar to the taxonomic diversity of CRISPR-containing contigs predicted in the HMP metagenome. In the DG metagenome, the majority of 16S rRNA sequences were assigned to Firmicutes and a smaller number, to Actinobacteria [45]. This breakdown generally matches the taxonomic breakdown of CRISPR-containing DG contigs with one exception: according to our data, one CRISPR-containing contig was assigned to Bacteroidetes. Probable reasons for these discrepancies might be biases in the estimation of the taxa abundance due to variability of the 16S rRNA genes copy number ranging from 1 to 15 per bacterial genome [60], or varying CRISPR prevalence in different bacterial phyla.

### CRISPR-*cas*types

Functional CRISPR-*cas* immune systems consist of CRISPR cassettes and *cas* genes [14]. We classified the identified systems according to repeat types and associated *cas* genes where possible. The latter were found in flanking sequences of 130 cassettes. In a large fraction of flanking sequences (50, 38%) the only identified *cas* gene was *cas1* (Additional file 2: Figure S2), a universal marker of all CRISPR-*cas* systems, hence not applicable for differentiating system types. Among cassettes that could be classified according to characteristic *cas* genes, 34 were assigned to CRISPR-*cas* type I; 25 cassettes, to CRISPR-*cas* type III; and 14 cassettes, to CRISPR-*cas* type II. For 29 cassettes, the composition of associated *cas* genes was sufficient to assign subtypes (Additional file 2: Figure S2, Additional file 1: Table S1).

CRISPR repeats may be divided into types based on sequence similarity and ability to form stable secondary structures [61, 62]. The repeat type is associated with certain *cas* genes, and hence the repeat sequence itself can be used as a classifying feature. Recently, an automated classifier of CRISPR repeats – CRISPRmap – was published [63]. CRISPRmap was designed for comprehensive classification of all known (i.e., publicly available) CRISPR cassettes based entirely on the repeat properties (sequence and secondary structure). Applying CRISPRmap to CRISPR repeats identified in the human gut microbiomes resulted in assignment of 191 unique repeat sequences corresponding to 233 cassettes to one of six superclasses (labeled A-F) (Additional file 1: Table S1). The representatives of all six superclasses were found. Superclasses F, E and D appeared to be the most populated ones. Of note, these superclasses contain families with little sequence conservation [63]. Repeats from 160 cassettes were not assigned with any superclass label according to the CRISPRmap classification; however, for 50 of these, a CRISPR-*cas* type could be determined according to the composition of the associated *cas* locus [62]. This suggests that these come from previously unknown CRISPR cassettes.

For 83 cassettes both classification labels could be assigned. For 17 cassettes the CRISPR-type assignments did not match the repeat-based classification (Additional file 3: Table S2). Contradictions were observed for only three repeat superclasses (F, C and D). This may indicate that the existing correspondence between *cas*-gene composition and repeat types should be revised.

### Identification of protospacers

We compared each of three non-redundant spacer sets with the metagenome it originated from. In the JPN non-redundant set, we identified 240 reliable spacer-protospacer pairs (Additional file 4: Table S3, Additional file 5: Figure S3). The observed protospacers corresponded to 136 different spacers (~5% of the JPN non-redundant set) and originated from 165 unique metagenomic contigs. For two metagenomic contigs (HumanGut_CONTIG_00179657 and HumanGut_CONTIG_00179696) the number of protospacers was remarkably high (16 and 10, respectively). These contigs clearly demonstrated a bacteriophage origin and were similar to *Bacillus* phi29-like phages. For one spacer from the JPN spacer set, we detected 19 protospacers, all of them coming from contig regions corresponding to bifidobacterial transposase genes. For 35 (15%) of non-redundant HMP spacers, we identified 89 spacer-protospacer pairs with protospacers coming from 59 different contigs. For the DG spacer set, no reliable spacer-protospacer pairs in the same metagenomic dataset were observed (Additional file 4: Table S3).

**Figure 3:**
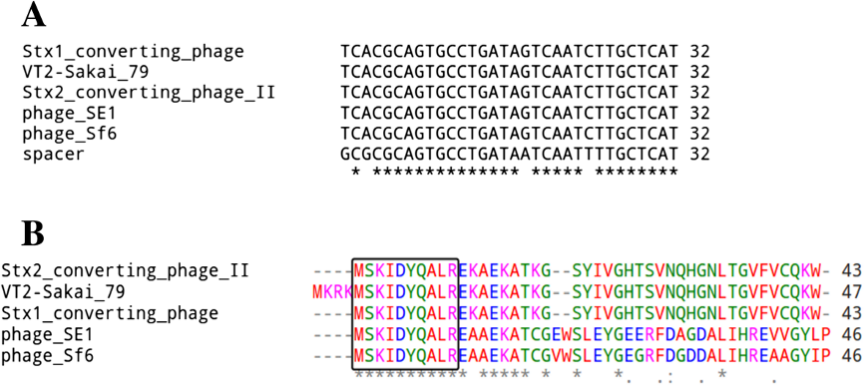
**The position of protospacer corresponding to the most conserved part of a protein related to the lambda phage** ***Ea22*** **protein in six related enterophages. (A)** Nucleotide sequence alignment for spacer and identified protospacers. **(B)** Amino acid sequence alignment for the respective protospacers. The protospacer position is shown by the black frame.

The comparison of the spacers with the NR collection of GenBank yielded 75 spacer-protospacer pairs for the JPN spacer set, corresponding to 17 spacers (Table 2). Among these spacer-protospacer pairs, eleven matches were found in complete viral genomes. The detected protospacers corresponded to four different spacers and were located in genomes of phages infecting *Escherichia*, *Salmonella*, *Clostridium* spp. and three unspecified enterobacteria (VT2-Sakai, epsilon15, Sf6). Notably, one of these spacers had protospacers, all with four mismatches, in five different enterobacterial phages: *Enterobacteria* phage VT2-Sakai, *Enterobacteria* phage Sf6, *Escherichia* Stx1 converting bacteriophage, Stx2 converting phage II, *Salmonella enterica* bacteriophage SE1. This protospacer corresponded to the most conserved region in the lambda phage protein *Ea22* gene, occurring in all five enterobacterial phages. This may reflect a close evolutionary relationship of these sequences with the real source of the spacer, and, probably, this spacer might mediate CRISPR-dependent multiphage resistance against a group of enterobacterial bacteriophages (Figure 3).

Seven spacers coming from contigs with the same repeat sequence matched protospacers in two unclassified phages; six residing in the unidentified phage clone 2204_scaffold14 (JQ680368.1) and one, in the unidentified phage clone 2209_scaffold1451 (Q680376.1). Both phages were isolated from human gut samples. Four protospacers originating from those unclassified phages occurred in coding sequences assigned to hypothetical proteins, while three protospacers were located in intergenic regions.

Three spacers matched protospacers in the complete genome of *Bifidobacterium longum* subsp. *infantis* 157 F. Remarkably, one of these protospacers was located in a gene encoding a putative phage tail protein (the ruler protein), i.e., it originated from a prophage sequence. The remaining two bifidobacterial protospacers originated from genes encoding conserved hypothetical proteins. Of note, the bifidobacterial protospacers corresponded to spacers from two CRISPR cassettes assigned to children.

The remaining three spacers for the JPN set matched protospacers of enterobacterial origin, residing in plasmids from *Escherichia coli*, *Salmonella enterica* and *Klebsiella pneumoniae.* One of the enterobacterial protospacers matched a plasmid gene encoding replication protein A from the *repFIB* replicon; another one matched a gene encoding a putative antirestriction protein, and the remaining protospacer corresponded to an intergenic region of various *E.coli* plasmids (pHUSEC41-1, pUMNK88_91, pND12_96, pECOED, pEK204, pEC_Bactec, pO113). Summing up, the majority of JPN protospacers found in GenBank sequences were of clear viral or plasmid origin.

Only nine protospacers, corresponding to nine spacers, were found in the NR collection for the HMP spacer set (Table 2). Of five protospacers matching regions in the genome of *Faecalibacterium prausnitzi*, two resided in intergenic regions, two corresponded to a gene annotated as the growth inhibitor protein, and the remaining protospacer matched CDS of a hypothetical protein. Three protospacers for HMP spacers matched sequences of uncultured organisms, clones LM0ABA27ZF12FM1 and VC1A546TR, both isolated from human gut samples. Finally, the remaining protospacer corresponded to a hypothetical protein-coding gene in *Bifidobacterium longum* subsp. *longum.*

No reliable protospacers were associated with the DG spacer set both in the same metagenomic dataset (Additional file 4: Table S3, Additional file 5: Figure S3) and among viral sequences from GenBank. The observed low number of matches with complete and partial phage genomes (deposited to GenBank) probably reflects the fact that most of the viral space remains unexplored.

In order to estimate the significance of detected protospacers, we performed a similar search against the NR collection for simulated pseudospacers (see Methods). For 2,992 spacers simulating the JPN spacer set, we detected 66 hits (mainly originating from multiple *E.coli* strains) corresponding to ten pseudospacer-pseudoprotospacer pairs. Unlike the real JPN spacers with identified protospacers, JPN pseudoprotospacers were mainly found in complete genomes of various bacterial taxa, corresponded to intergenic regions, and showed no tendency to match mobile elements or genes associated with prophages or viruses. Based on this simulation, we posit that protospacers corresponding to spacers from CRISPR cassettes are not random matches obtained by chance and are indeed associated with defence against mobile elements such as viruses and plasmids. Similar results were obtained for the HMP and DG spacer sets (data not shown).

Although short protospacer adjacent motifs (PAMs) are considered to be a common feature of many diverse CRISPR systems [58, 64], we could not detect any reliable PAM motifs for the protospacers clustered by the repeats.

### Taxonomy of protospacer origin and compatibility with the CRISPR-cassette taxonomy

The taxonomy of CRISPR-containing metagenomic contigs can be determined relying on either flanking sequences or protospacers. When a metagenomic contig was assigned with both types of taxonomic labels, they were compared.

Out of 296 CRISPR-containing contigs in the JPN metagenome, 73 had taxonomy status assigned by the flanking sequences, 13 contigs had taxonomic labels based on the protospacers and, seven had both types of taxonomic labels. Five of them demonstrated a good concordance between the taxonomic labels, as the assignments agreed at least on the level of phyla. For two contigs with an uncertain flank-based assignment (“Bacteria”), the protospacer-based taxonomy was more specific (Additional file 6: Table S4).

Out of 78 HMP contigs with CRISPR cassettes, 48 were assigned with taxonomic labels based on the taxonomy of cassette-flanking regions, and for six contigs the taxonomic assignment could be made according to protospacers. Only three contigs had both taxonomic labels, and in all cases they were in a general agreement (Additional file 6: Table S5).

### Similarity of the spacer composition in the human gut microbiomes

A pair of adjacent spacers was observed in more than one metagenome, in the JPN and HMP datasets. The respective contigs overlapped by a region containing these two spacers and a short flanking sequence of 134 nt. According to the independently assigned taxonomic labels, both cassettes were of the same taxonomic origin, belonging to Firmicutes.

**Figure 4:**
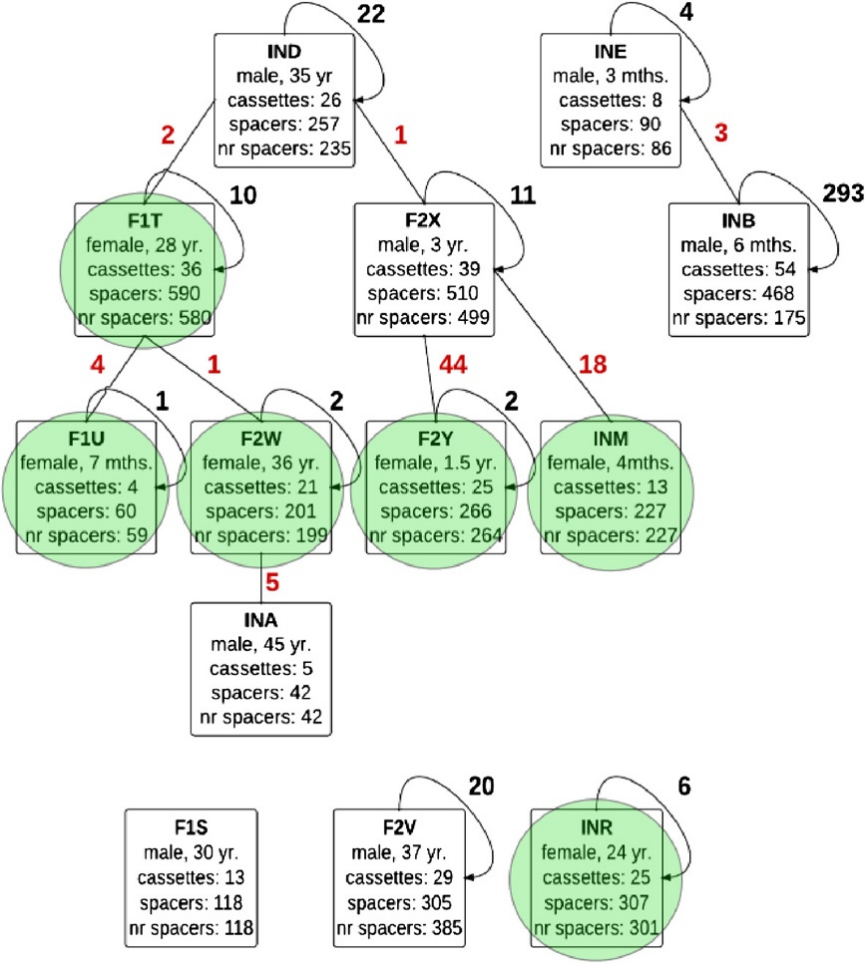
**Shared spacers between JPN individuals.** Each square stands for an individual metagenome (male – blank square, female – square with a green circle). Identifier for each individual, gender, age, the numbers of predicted cassettes and spacers are specified within the respective square. Individuals with identifiers starting with ‘F’ belong to families (F1 and F2), individuals with identifiers starting with ‘IN’ are independent. Numbers of shared spacers are shown on the edges connecting individuals; numbers of repeating spacers in the same individual are shown on the directed edges.

In individual JPN metagenomes, the largest number of shared spacers was observed between CRISPR cassettes assigned to children, in particular, for the F2X-F2Y and F2X-INM pairs (44 and 18, respectively). In three pairs (INE-INB; F1T-F2W; F2W-INA), complete CRISPR cassettes with flanking sequences were shared. In almost all cases, shared spacers originated from CRISPR cassettes with identical direct repeats, the only exception being the F1T-F2W pair having one mismatch between the repeat sequences (Figure 4).

The number of spacers occurring in more than one individual (shared spacers) in the JPN metagenome was 78. To check whether this is significantly more or less than expected at random, we applied a shuffling procedure. At that, we randomly replaced cassettes between individuals, so that the number of cassettes assigned to a given individual did not change. This procedure was applied 100,000 times, and the number of shared spacers was calculated. The average number of shared spacers was 127, and the distribution is shown in Additional file 2: Figure S4. In all cases the number of shared spacers was larger than the observed one, indicating that the latter was significantly below the random expectation (*p <* 10^-5^).

To estimate the CRISPR core, we considered the distribution of repeats among individual metagenomes. No universal or widely distributed repeat clusters (indicating the same or similar cassettes) were detected (Additional file 2: Figure S5, Additional file 6: Table S6). The overwhelming majority of repeat clusters (290) appeared to be highly specific and associated with only one particular individual. Still, 24 repeat clusters were shared between at least two individual metagenomes. The most widely distributed repeat cluster was found in five different individuals (all from the JPN metagenomic dataset). At that, four repeat clusters were found in individuals coming from different metagenomic datasets (HMP and JPN), i.e., geographically distant populations, indicating a possibility for a common CRISPR core in the human gut metagenomes. However, available data were insufficient to further address this problem.

### Spacer-protospacer co-occurrence in individual metagenomes

We then analyzed whether protospacers have a tendency to originate in the same individual metagenome as the spacers (Additional file 5: Figure S3, Additional file 4: Table S3). We analyzed the combined human gut set that contained 139 individual metagenomes. 41 (32%) of individuals had a majority of protospacers originating in other individual metagenomes. For 37 (28%) of such individuals there was one particular individual metagenome that contained a large fraction of protospacers. The preference of spacers to have protospacers in the same individual metagenome was clearly observed in the F2Y, INB and INR individuals, featuring a considerable number of spacer-protospacer pairs (26, 49 and 14, respectively). No cross-matching pairs between siblings (F2X and F2Y) were observed (Additional file 4: Table S3). Surprisingly, we identified a large number of protospacers in the JPN dataset that corresponded to spacers originating from the HMP dataset, much more than in the HMP dataset itself. A possible explanation comes from the HMP metagenomic DNA purification protocol: the procedure included filtering of a sample suspension through 100 um mesh nylon membrane [43]. This procedure probably eliminated viral particles and subsequently viral sequences in the HMP metagenome, further leading to the relative scarcity of HMP protospacers for HMP spacers, compared to JPN protospacers. The fact that we observed many spacer-protospacer pairs in different individuals suggests that viruses associated with the human gut are to some extent universal, containing a fraction of ubiquitous viruses present in many individuals.

To check whether spacers and protospacers tend to co-occur, the CMH statistic was calculated for the real data and simulated data (see Methods). The CMH value for the observed data was 5.18, while the distribution of CMH statistic for the shuffled tables is shown in Additional file 2: Figure S6. In the majority of cases the actual CMH statistic was larger than that calculated for the shuffled tables, (*p*-value = 0.182), however, not reaching significance.

The observed lack of clear preference of spacers and protospacers to occur in the same individual suggests that CRISPR systems in most individual metagenomes are active and highly effective against bacteriophages. A similar observation was made earlier [39]. On the other hand, in several studies focusing on CRISPR dynamics in human oral microbiomes [35–37], protospacers for the respective spacers were more likely to be identified when the oral virome of the respective individual was also available. So, the observed scarcity of identified protospacers for the gut CRISPR spacers may be revised when individual gut viromes will be also available for comparison.

Compared with the spacer-protospacer co-occurrence patterns in CRISPR systems from the ocean metagenome [26], the CRISPR composition of the human gut seems to be more homogeneous as some spacers happen to have protospacers in geographically distant populations. It is a likely consequence of the relatively larger stability and uniformity of environmental conditions in the human gut (temperature, pH, salinity, *etc*.), compared to the physical and chemical characteristics of the ocean samples. The latter differ dramatically, so their CRISPR content does as well.

### Position of targeting spacers and shared spacers

**Figure 5:**
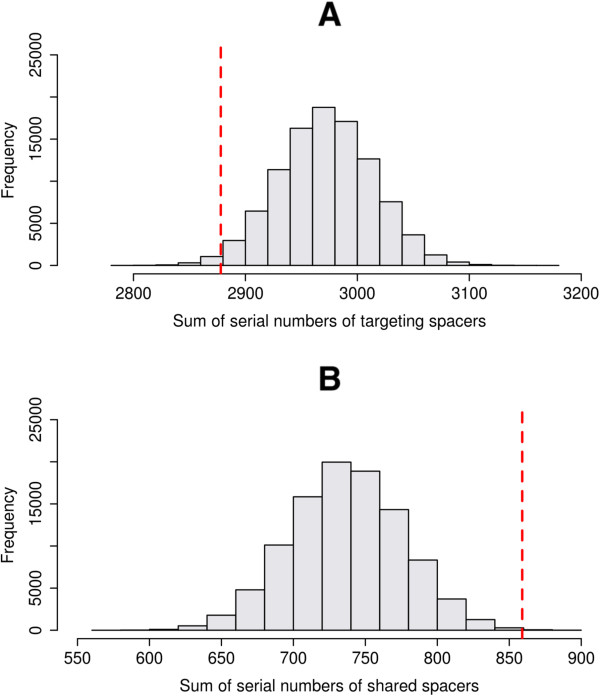
**Positions of functionally important spacers compared to positions of all spacers.** Targeting **(A)** and shared **(B)** spacers are presented. Spacers in each cassette were enumerated. For each cassette, serial numbers of all targeting (resp., shared) spacers in the cassette were summarized, and then the obtained statistics was summarized for the whole set of cassettes (the red dashed line). The plot shows the distribution of sums of serial numbers of targeting (resp., shared) spacers for the shuffled sets of cassettes (100,000 permutations) (see Methods).

Positions of targeting spacers, i.e., spacers having protospacers in the same individual, are shifted to the leader end of a cassette (*p*-value < 0.0002) (for details see Methods) (Figure 5A), whereas spacers shared between metagenomes of different individuals tended to be located close to the trailer end of a cassette (*p*-value < 0.001) (Figure 5B), and hence are older.

These observations agree with earlier reports about selected, experimentally studied systems. Indeed, in response to the phage infection, Streptococcus thermophillus alters its CRISPR loci by adding new spacer-repeat units to the leader end of the cassettes [18, 19]. Further, reconstruction of CRISPR loci of an extremophilic archaeon, I-plasma, showed that the leader-end spacers are highly diverse while the trailer-end spacers tend to be clonal population-wide [65]. The observed clonality of the trailer ends could be caused by consecutive selective sweeps that would homogenize CRISPR composition in a population, followed by differential addition of new spacers at the leader end.

## Conclusions

We analyzed CRISPR systems in the metagenomic datasets of three human gut microbiomes. A detailed comparison with other studies [38, 39] demonstrated complementarity of read-based and contig-based approaches — while many spacers remained in unassembled reads, our contig-based procedure could identify cassettes with new types of repeats and characterize the evolutionary dynamics of spacers within cassettes.

In all three metagenomes, the largest fraction of contigs with CRISPR cassettes was assigned to Firmicutes. Comparison of the identified spacers with the GenBank NR collection and complete viral genomes yielded only few matches, revealing that the viral space remains largely unexplored. Contrariwise, we found an appreciable number of spacers originating in the same metagenomic datasets. Based on the implemented statistical analysis, we could not reject the hypothesis that the observed co-occurrence of spacers and their protospacers was generated by chance, and moreover a considerable fraction of spacers had protospacers originating in other gut metagenomic datasets.

On the other hand, the overlap in spacers between different human gut metagenomic samples was negligible. Here we encounter an apparent paradox: a low spacer similarity between individuals versus a considerable number of spacer-protospacer pairs originating in different individuals. The absence of spacer-protospacer pairs in the same individual may result from high efficiency of CRISPR systems against viruses, i.e., fast elimination of the respective bacteriophage from the environment [39].

On the other hand, the occurrence of protospacers in other individuals suggests that there exists a common viral core inhabiting the human gut, and that this core may be shared between geographically distant populations. The extent to which CRISPR systems acquired resistance against these common viruses, i.e., the presence of spacers, may vary between individuals.

In comparison with CRISPRs in the ocean metagenome [26], the human gut appeared to be a more homogeneous environment as some spacers have protospacers in geographically distant populations. A possible explanation comes from the uniformity of environmental conditions in the human gut compared with different ocean samples (temperature, pH, salinity, *etc*.). A broad range of physical and chemical conditions in the Ocean results in variability of the microbial and phage composition, and, consequently, differences in the CRISPR content.

The contig-based approach allowed us to reconstruct the order of spacers in CRISPR cassettes. Targeting spacers tend to be located closer to the leader end. As this is the site of addition of new spacers [18, 19], this indicates a footprint of recent bacteriophage infections. *Vice versa*, spacers shared between individual metagenomes tend to be located closer to the trailer-end of the cassettes, and hence represent a more ancient, common state of the CRISPR based immunity [62, 66].

## Electronic supplementary material

Additional file 1: Table S1: Characteristics of reliably predicted CRISPR cassettes. Classification of adjacent *cas* genes, where possible, and CRISPRmap classification of repeat sequences are provided. Background colors denote samples (yellow – DG, red – HMP, blue – JPN). (XLS 96 KB)

Additional file 2: Figure S1: CRISPR detection in relation to metagenome characteristics. Vertical axes: numbers of identified cassettes (A, C) and spacers (B, D). Horizontal axes: average contig length (A, B) and total contig length (C, D). Each dot corresponds to an individual human gut metagenome: red dots represent DG individuals; blue dots, JPN individuals; green dots, HMP individuals. **Figure S2.** Distribution of CRISPR-*cas* types in the identified cassettes. The classification is based on the *cas-*loci composition, see the text for details. **Figure S4.** Distribution of the number of shared spacers between individuals for 100,000 random permutations. The red dashed line shows the number of observed shared spacers. **Figure S5.** Distribution of repeat clusters across individual metagenomes. **Figure S6.** Distribution of the CMH statistic for independence of spacer and protospacer occurrences in individual metagenomes. The distributions are based on 10,000 permutations of protospacers across individuals (for the simulation details see the text). The red dashed line shows the observed CMH statistic. (PDF 144 KB)

Additional file 3: Table S2: Compatibility of the CRISPRmap classification of repeat sequences and the CRISPR-Cas system classification of adjacent *cas* genes. Inconsistent assignments are highlighted. (XLS 24 KB)

Additional file 4: Table S3: Number of spacer-protospacer pairs per individual. Columns – metagenomes containing spacers, rows – metagenomes containing protospacers; each cell shows the number of spacer-protospacer pairs originating in the respective metagenomes. Background colors in the header columns and rows denote samples (yellow – DG, red – JPN, blue – HMP). Cells have green background if the respective value is non-trivial (>0) or grey background if the spacer and protospacer originate in the same sample. (XLS 132 KB)

Additional file 5: Figure S3: Heatmap of the spacer-protospacer pairs distribution between individual metagenomes. Colors reflect the numbers of detected pairs (shown in the heatmap). (PNG 310 KB)

Additional file 6: Table S4: Comparison of taxonomic labels assigned to CRISPR-containing contigs and respective protospacers for the JPN metagenome. Column ‘protospacer’ shows the taxonomic label of the contig containing the respective protospacer; column ‘contig’ shows the taxonomic label of the contig containing the respective cassette. **Table S5.** Comparison of taxonomic labels assigned to CRISPR-containing contigs and respective protospacers for the HMP metagenome. Column ‘protospacer’ shows the taxonomic label of the contig containing the respective protospacer; column ‘contig’ shows the taxonomic label of the contig containing the respective cassette. **Table S6.** List of shared repeat clusters. All repeats observed in at least two individuals are listed. (XLS 16 KB)

## Abbreviations

bp: Base pairs
cas(Cas): CRISPR-associated
CRISPR: Clustered regularly interspaced short palindromic repeats
crRNA: CRISPR RNA
CMH: Cochran–Mantel–Haenszel test
DG: Distal gut metagenomic project
GOS: Global ocean sampling expedition
HMP: Human Microbiome Project
JPN: Metagenome of 13 healthy japanese individuals
NR: Non-redundant
PAM: Protospacer-adjacent motif
VLP: Virus-like particles.
