# Supplementary material for: Comparative analysis of CRISPR cassettes from the human gut metagenomic contigs
Source: BMC Genomics. 2014 Mar 17;15(1):202. doi: 10.1186/1471-2164-15-202 (PMC4004331; doi:10.1186/1471-2164-15-202)

Supplementary Figure S1

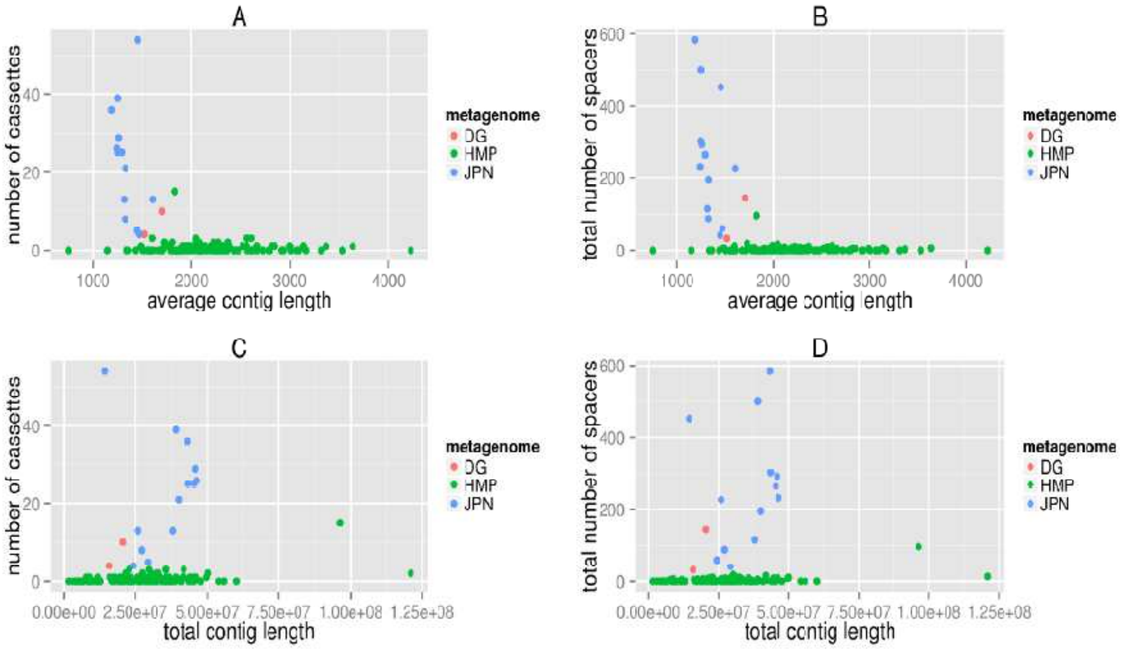

Supplementary Figure S2

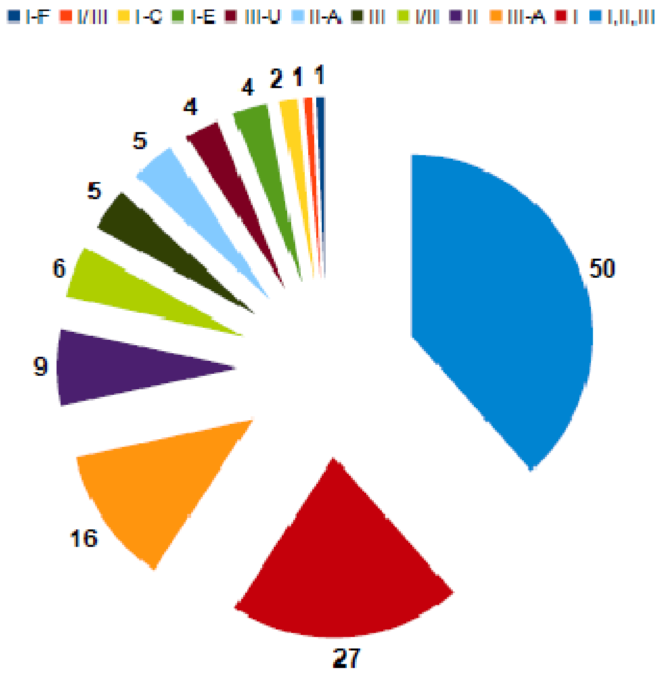

Supplementary Figure S4

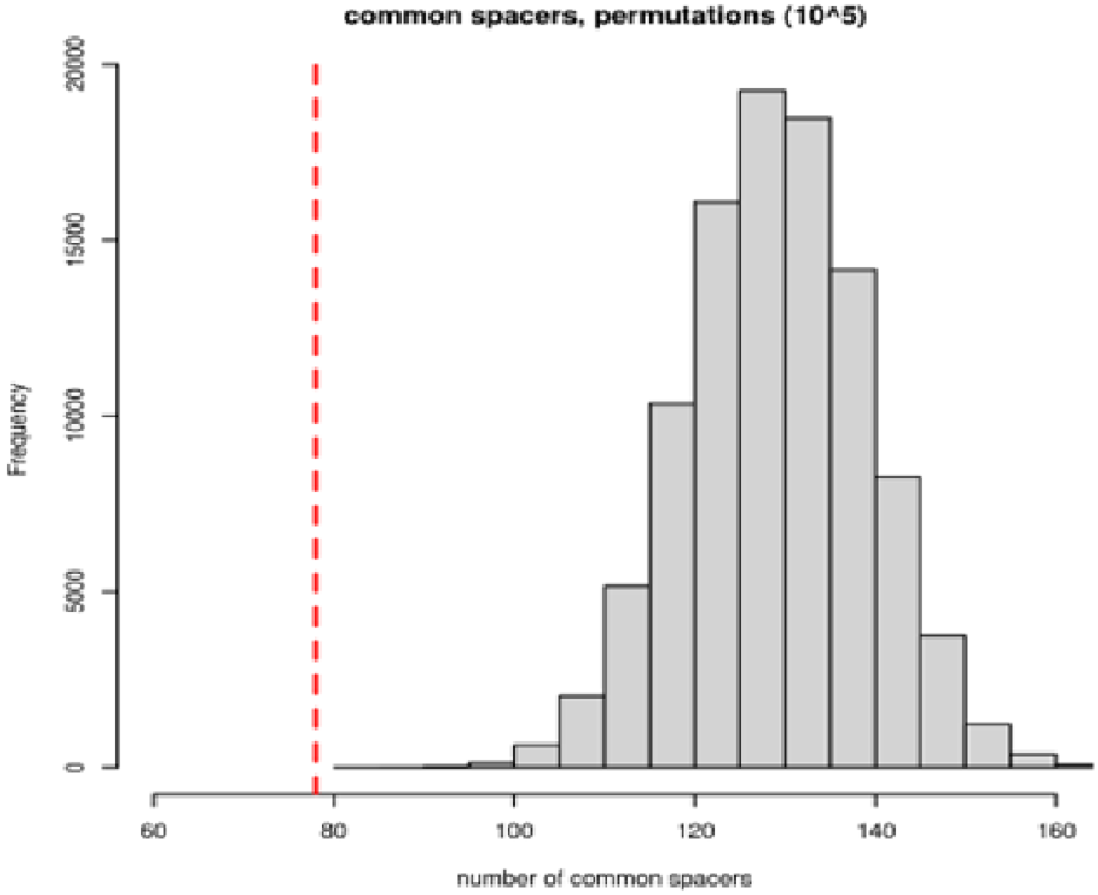

Supplementary Figure S5

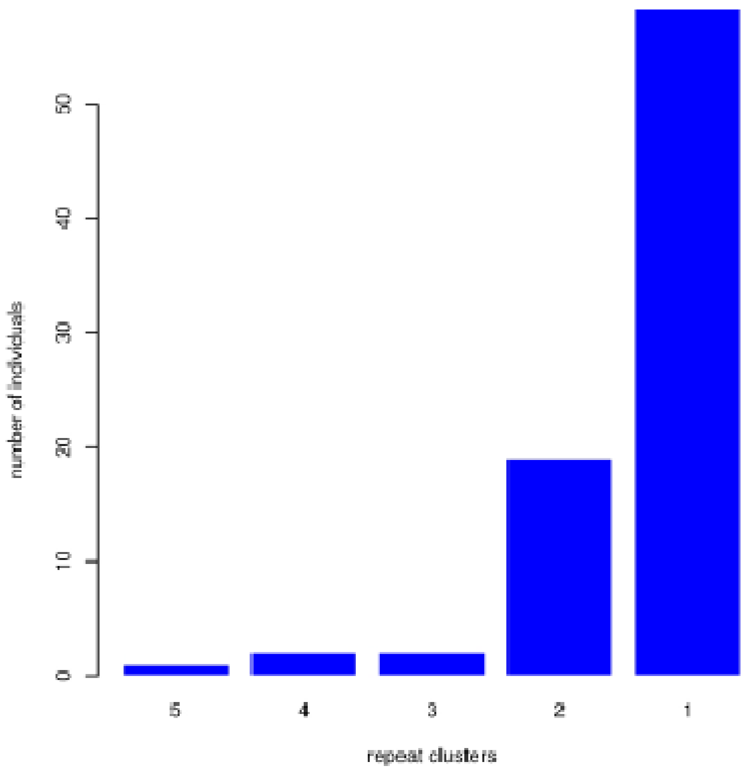

Supplementary Figure S6

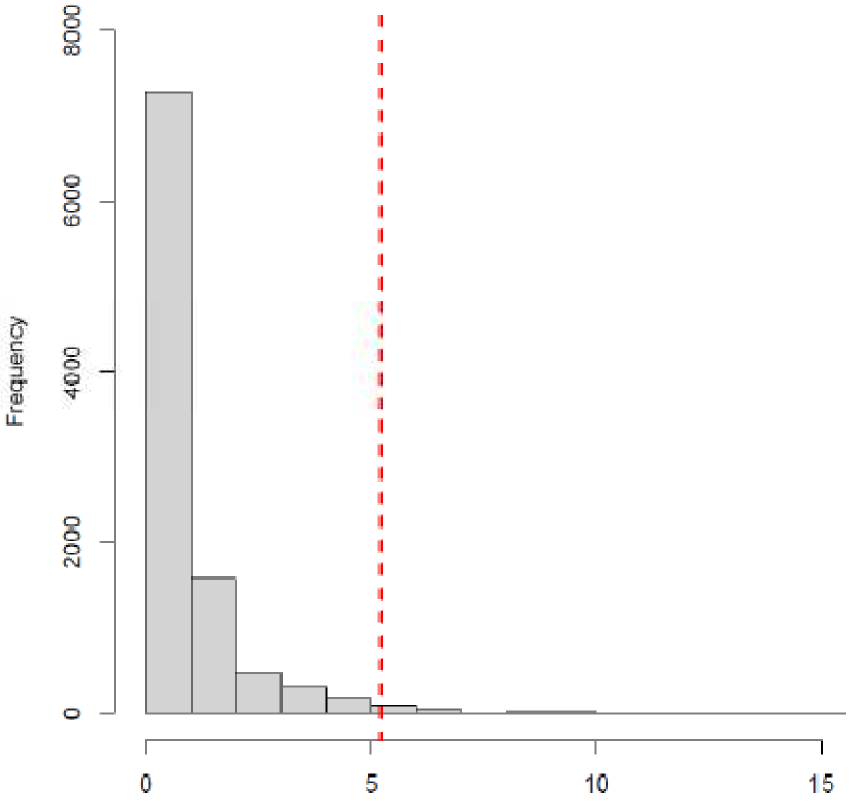

Supplement: Supplementary file 2 — Additional file 2: Figure S1: CRISPR detection in relation to metagenome characteristics. Vertical axes: numbers of identified cassettes (A, C) and spacers (B, D). Horizontal axes: average contig length (A, B) and total contig length (C, D). Each dot corresponds to an individual human gut metagenome: red dots represent DG individuals; blue dots, JPN individuals; green dots, HMP individuals. Figure S2. Distribution of CRISPR-cas types in the identified cassettes. The classification is based on the cas-loci composition, see the text for details. Figure S4. Distribution of the number of shared spacers between individuals for 100,000 random permutations. The red dashed line shows the number of observed shared spacers. Figure S5. Distribution of repeat clusters across individual metagenomes. Figure S6. Distribution of the CMH statistic for independence of spacer and protospacer occurrences in individual metagenomes. The distributions are based on 10,000 permutations of protospacers across individuals (for the simulation details see the text). The red dashed line shows the observed CMH statistic. (PDF 144 KB) [file 12864_2013_7040_MOESM2_ESM.pdf]
