# Supplementary figures and images for: Comparative analysis of CRISPR cassettes from the human gut metagenomic contigs
Source: BMC Genomics. 2014 Mar 17;15(1):202. doi: 10.1186/1471-2164-15-202 (PMC4004331; doi:10.1186/1471-2164-15-202)

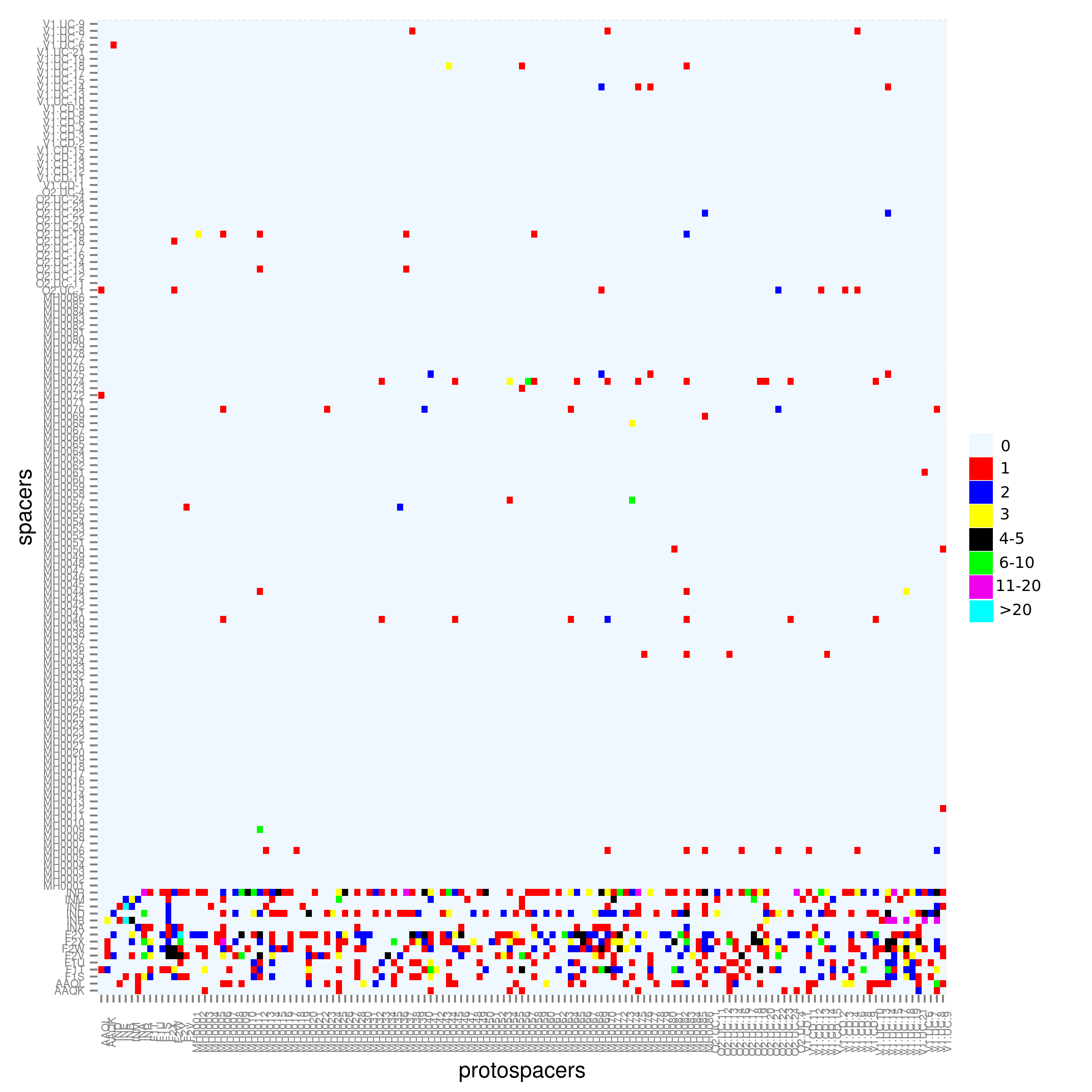

Supplement: Supplementary file 5 — Additional file 5: Figure S3: Heatmap of the spacer-protospacer pairs distribution between individual metagenomes. Colors reflect the numbers of detected pairs (shown in the heatmap). (PNG 310 KB) [file 12864_2013_7040_MOESM5_ESM.png]
